# Mini-Review of Biofilm Interactions with Surface Materials in Industrial Piping System

**DOI:** 10.3390/membranes13020125

**Published:** 2023-01-18

**Authors:** Haoyi Yang, Zezheng Xu, Zetong Xu, Yuanzhe Li

**Affiliations:** 1 NUS College of Design and Engineering, National University of Singapore, Singapore 118429, Singapore; 2 UNSW Environment Leadership Program, The University of New South Wales, Kensington, NSW 2052, Australia; 3 Qingdao Huanghai Vocational Institute, Qingdao 266555, China; 4 School of Materials Science & Engineering, Nanyang Technological University, Singapore 639798, Singapore

**Keywords:** bacteria interactions, biofilm attachment, surface materials, industrial piping system, biofilm removal

## Abstract

The growth of biofilm, which is caused by microorganism accumulation and growth on wetted surfaces, may damage industrial piping systems, increase maintenance and cleaning costs for the system sterilization, and even divulge the immune system into high risk. This article systematically analyzes the biofilm interactions with piping surface materials from the perspectives of physical convection, and biological and chemical adhesion. The thermodynamics of the flow, bacterial surface sensing, and bio-communication are the most critical factors for biofilm attachment. Furthermore, experimental analysis methods as well as biofilm control and removal approaches, are also included in this study. Finally, the resistance and growth of biofilm, as well as the practical and advanced methodology to control the biofilm and challenges associated with technology, are also discussed. Moreover, this paper may also offer a significant reference for the practice and strategic applications to address the biofilm resistance issues in industrial piping.

## 1. Introduction

Biofilms are the dominant life form on earth. More than 99% of all microorganisms and more than 90% of all organisms occur as microbial aggregates [1]. They are found in numerous natural and engineered systems. Therefore, pathogen biofilms are the main reason for bioremediation in most water purification systems, industrial processes, and metal ship hulls, etc. Biofilm is an aggregation of microorganisms, which include cells, bacteria, etc., at an interface plus extracellular polymeric substances (EPS) [1,2]. In the industrial pipeline system, the planktonic microorganisms are far more numerous and, also, more important for biological transformations than the biofilm cluster. Their abundance in industrial piping and their role in carbon turnover far outweighs that of biofilm organisms [2,3].

**Figure 1:**
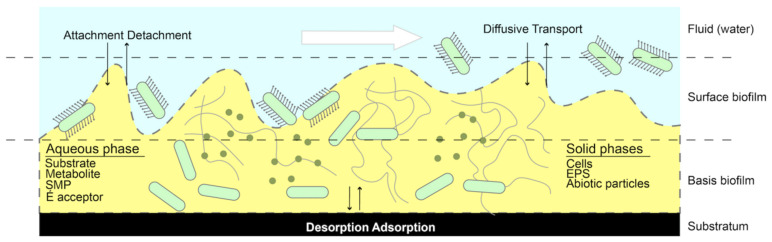
Schematic diagram of biofilm development in the material surface of piping system.

By forming a conditioning layer, the biofilm substrate may adsorb at the interface, thus making it attractive for planktonic bacteria. It is composed of a layer of protein that is deposited by a protein found in the environment or the bacteria itself [4]. Higher concentrations of nutrients on surfaces may contribute to organic material settling and depositing on surfaces, e.g., the *Caulobacter Crescentus* may exploit the exterior attachments to improve nutrient absorption [5]. Moreover, the biofilm substrate will use metals (e.g., magnesium and iron) as an ultimate oxidant (e.g., *Shewanella* and *Geobacter Sulfurreducens* use *pili* to transport ions between cells and surfaces) [6]. Such a conditioning layer process can mask the underlying functional groups that reduce cell adhesion on the piping surfaces (e.g., quaternary ammonium salts) [7]. Moreover, swarming and quorum sensing are another two significant factors for the initial attachment of the bacteria and biofilm interaction process with the surface. Swarming starts from a precursor of surface sensing. It is able to reduce susceptibility to antibiotics and mutualistic interactions among the biofilm clusters [8,9], whereas the fluctuation in cell population and density responds to quorum sensing (QS) [10]. Quorum sensing improves communication in high-density cells using small-molecule chemical messengers. It can transfer information between cells to trigger physiological changes as well as the lateral gene in biofilms. Then the bacteria adhere to the interface and multiply [11]. The thickness of the ensuing biofilm is influenced by growth and detachment processes. Detachment may occur as a result of mechanical shear caused by fluid movement inside the pipe system, which can lead to erosion, sloughing of cells and entire biofilm chunks, abrasion, and dispersion (Figure 1) [12]. However, this detaching process may also boost the biofilm’s antibiotic resistance which includes (i) dormant persister cells, (ii) barrier function of biofilm matrix, and (iii) upregulation of biofilm-specific antibiotic resistance (ABR) genes [13]. Moreover, the protection from predators (e.g., *Serratia marcescens* and *protozoa*) also increases [14].

Biofouling is a significant and costly issue for the industry. If the mature bacterial biofilm is allowed to grow unabated in towers cooling, circuits refrigeration, regenerators, receptacles, and any other industrial piping system, it will quickly spread in all directions [15,16,17]. When the corresponding biofouling platelets approach a certain peak, the current rips out into pieces of biomass. After that, the water is moved in the flow direction. The microorganism development on reverse osmosis and ultrafiltration membranes in desalination, and water treatment facilities may be the source of this torn biofouling’s settlement someplace else in the water system and the beginning of a new biofilm creation. A number of sectors are negatively impacted by biofilm growth, including those that deal with food and water contamination, greater bacterial immune system resistance, and higher cleaning and maintenance expenses [18,19].

In this paper, the bacteria and biofilm interactions with piping surface materials in wastewater treatment plants have been analyzed systematically from the perspectives of (i) bacteria/biofilm and surface interaction mechanisms study, and (ii) cell attachments to different piping materials. In the meantime, practical maintenance methodologies and potential risks are also mentioned. Positional perspectives on how to approach the problem of biofilm resistance in these industrial piping systems have also been raised in detail [20].

## 2. Mechanisms Study of Biofilm and Surface Interaction

### 2.1. Cell Attachment Mechanisms

Reversible attachment and irreversible attachment are the two stages of bacterial adhesion to a surface [13].

#### 2.1.1. Reversible Attachment

Reversible attachment transpires within a minute after hydrodynamic and electrostatic interaction with the piping material facet being developed [9]. Bacteria can detach and return to their planktonic state within this brief time because it is temporary and reversible [19,21]. However, due to physicochemical effects, such as the disruption of the water boundary, the introduction of fundamental diversity in the exterior fragment, and the modification of the body cell to develop a connection to the surface, such a process may also quickly increase the adhesive forces between bacteria and surfaces. Throughout the amendable attachment, the pathogen microorganism either utilizes nanoporous (e.g., *flagella* and *pill*) along the proximity of adhesins and the production of EPS to connect between the body cell and infrastructure that accelerates adhesive forces [22]. Other than that, the negative and positive charge interaction may also contribute to the reversible attachment process that has a maximum pathogen that has both negative and positive net that interact on a charged surface. Thus, quorum sensing would increase the negative charge on cell surfaces (e.g., *E. coli*) as well [23]. 

The connected pathogen develops and reproduces, ignited by supplements in the H_2_O, and ejects a viscous, gel-like, adherent material known as EPS which is made up of natural polymers of high molecular weight secreted by microorganisms as well as cellulose matrix. The cellulose matrix is able to fix the development of a biofilm to the exterior, whereas, the polysaccharide also plays an important role in the foremost attachment of the organism and activity as a bond during the process [9].

#### 2.1.2. Irreversible Attachment 

Irreversible adherence happens gradually outside within hours after the reversible process [14]. Irreversible attachment involves Van der Waals interactions that occur at the plasma membrane cooperating with the inner cytomembrane at a distance [1,19]. This results in adhesins that expose the embryo surface to develop a “key-lock” joint between the cells and the anchorage with the support of amino acids. The production of EPS may also anchor cells to piping surfaces. Lipopolysaccharides (LPS) and fiber inflate the rate of microorganism attachment (e.g., *E. coli*). The modification of chromosome formulation would initiate by revolving the nuclear system approaching biofilm layout. As a result, the mechanoreceptor produces signal transduction halfway sensing and retort to forces constructed by the surface attachment. Some proteins serve as antibiotic resistance as well as secreted proteins (e.g., *Pseudomonas fluorescens*) during the process. This indicates that through irreversible attachment, pathogens would have conquered the dreadful forces of the mechanical binary layer in the reversible attachment tribune, and started a chemical take when in contact with a lattice of the same conditioning layer [24]. 

### 2.2. Experimental Technologies of Industrial Biofilm Interaction Observation

The research methods have been limited to material recycled to investigate microbe-surface intercommunication and translated usage of broad biocompatible substances (studying mammalian cell attachments to surfaces) for bacterial cell attachment and growth [25]. The current methodology to measure adhesion between bacteria and a surface may generally be performed as described below:

#### 2.2.1. Bacterium Attachment to Hydrocarbons (MATH) Analysis

To assess the degree of cell attachment to hydrophobic liquids and/or the hydrophobicity of microbial cells, the microbial adhesion to hydrocarbons (MATH) experiment has been frequently utilized. The MATH test has assisted in elucidating surface structures that promote hydrophobins or reduce the hydrophobic properties of various microorganisms’ outermost cell surfaces [26]. In the standard MATH assay, the initial and final cell concentrations in an aqueous cell suspension that has been in contact with a hydrocarbon liquid are measured using spectrophotometric absorbance. In contrast, the cutting-edge technology of microscopic analysis of the aqueous suspension and direct cell counts produces cell concentrations devoid of hydrocarbon droplet interference. In MATH tests carried out with bacterial strains, hydrocarbon droplets were seen [25,26].

#### 2.2.2. Atomic Force Microscopy (AFM)

Atomic force microscopy (AFM) was used to examine the nanoscale surface morphology of bacteria-mineral aggregates and biofilms produced on clay-sized minerals, as well as the adhesion forces between bacteria and goethite in water. Polymeric-based surface elasticity of industrial piping has previously been shown to have a strong influence on bacterial adhesion; thus, the AFM recently has been applied to investigate the bio-fouling potential of membranes, as well as the surface elasticity [26]. AFM technologies that can be utilized to understand separation processes at the membrane surface now include the novel use of AFM to quantify Young’s moduli and work of adhesion on membranes [27]. AFM force spectroscopy can also be utilized as a component of an advanced membrane autopsy technique to clarify the mechanisms underlying membrane and bio-fouling, according to studies.

#### 2.2.3. Total Internal Reflection Microscopy (TIRM) Illuminator

Total internal reflection microscopy (TIRM) illuminator is a high-contrast imaging technique, as well as a quantitative tool for the measurement and study of colloidal forces and biological events that take place on or near the cell membrane [28]. The advanced TIRM illuminator may even improve its contrast by restricting the thickness of the excitation field to over an order of magnitude narrower than an epi-fluorescence microscope’s z-resolution and signal-to-noise ratio, making it a useful tool for imaging cellular events such as vesicle exocytosis or endocytosis, viral particle formation, cell signaling, and membrane protein dynamics [29]. The TIRM system can be used to reduce illumination field aberrations, to quickly switch between epi-illumination, and to adjust penetration depth during multicolored applications [30].

#### 2.2.4. Quartz Crystal Microbalance (QCM)

Regarding the biofilm characterization methodology of the industrial wastewater samples by the third-party verifiers or labs, Quartz crystal microbalance (QCM) is the most widely used and popular acoustic transducer for sensor applications. Because of its high sensitivity, robustness, small size, and ease of integration with electronic measurement systems, it has found widespread application in chemical and biosensing fields. QCM was used to examine the fouling of the graft layers in real time after incubation in cell culture medium, fibronectin, and fetal bovine serum (FBS) solutions. However, QCM must be coated with a sensing film. Its selectivity and sensitivity are not obtained without the use of coating materials [31]. However, this is no longer an issue, owing to advancements in oscillator circuits and dedicated measurement circuits.

## 3. Cell Attachments to Different Surface Materials in the Industrial Piping System

**Figure 2:**
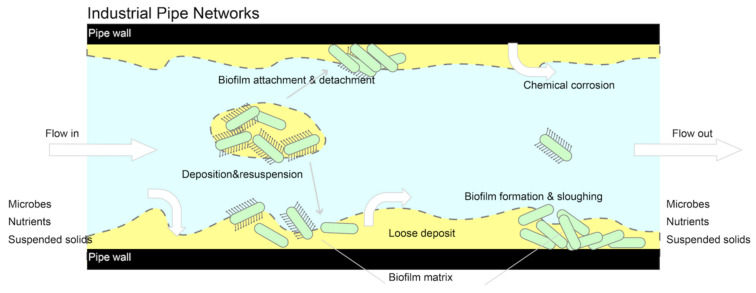
Schematic diagrams of microbial contamination and resistance in industrial piping networks.

On both biotic and abiotic pipe surfaces, biofilms can form with subsequent adherence depending on the characteristics of both the bacteria and the substrate. The process of bacterial attachment and biofilm formation typically starts within the first 24 h with (i) the pre-conditioning of the adhesion surface through the adsorption of suspended particles and organic species from the bulk fluid, (ii) the transport and attachment of the planktonic cells, and (iii) microbial multiplication and EPS production, which may depend on various factors such as population density, nutrient status, species composition, etc. [32]. The external mass transfer resistance is significant in the industrial piping system (Figure 2) because it can exacerbate oxygen or nutrient limitation in biofilms, worsen product inhibition, obstruct quorum sensing, and encourage the growth of tall, finger-like biofilm clusters. Examples of mass transfer that can produce extra force components include eddies, vortex streets, turbulent wakes, and turbulent bursts [4,5,6,7,33]. Additionally, Table 1 highlights the placements of various attachment processes as well as the preferred biofilm according to the biofilm development period. The following chapters and parts [32,33,34,35] will provide a full explanation of the niche process analysis.

### 3.1. Biofilm Stress Response

#### 3.1.1. Thermodynamics and Convection

Under slow water flow rates, the shear force was relatively low, allowing biofilm to grow quickly because fluid could accord the diffusive mobility of the surrounding environment and the metabolic substrate. Oxygen, proteins, and any other important substance for biofilm cell growth were provided [36]. However, due to access, such rapid growth was restricted to the top of the biofilm, and these regions expanded preferentially. The different shear force and convection influences of both hydrophobic and hydrophilic piping surfaces could lead to the following conclusions [24,37]. The slow-moving fluid that is close to the biofilm prevents solutes from diffusing into and out of the biofilm. Biofilm cells will cluster when fluid travels around them but not through them [12,36]. The convection, however, can still be complicated around the heterogeneous structures of a biofilm, i.e., once moving fluid exerts forces on the biofilm, such convection and stress response can result in biofilm deformation and movement by stretching, rolling, and rippling, and high fluid forces can even cause the biofilm to separate from the substrate [38].

#### 3.1.2. Surface Energy and Wettability

Surface energy and roughness play a major role in determining a surface’s inherent wettability. Different models have been used to explain the connection between surface energy/interfacial interaction energy and bacterial adherence. These models are created using Derjaguin-Landau-Verwey-Overbeek (DLVO), extended-DLVO, and thermodynamics-based methods. For instance, a thermodynamic approach based on the pairwise interaction of surface-free energy among surface, fluid, and bacteria was utilized to explain bacterial adhesion processes. In terms of hydrophilic materials with high surface energies, bacteria have surface energies that are higher than those of liquids, and their surface microstructure is primarily concave, or curved inward [7,39]. The biofilm and the surface materials exhibit high electrostatic interaction and cohesiveness [22,39]. On the concave of the surface microstructure, microcolony development and biofilm maturation become unquestionably irreversible. Additionally, the decomposing biofilm cells might be used as raw EPS materials to draw in new planktonic cells inside the pipe system. While bacteria’s surface energy is unquestionably lower than that of liquids when compared to hydrophobic materials with lower surface energies, the hydrophilic biofilm cluster can fail even more easily than the hydrophobic one by triggering a detachment event due to the opposite trend of electrostatic interaction. Additionally, according to morphological observations, hydrophilic surfaces may have more convex surface microstructures than hydrophobic surfaces which curve outward. In conclusion, hydrophobicity and hydrophilicity are preferred for the attachment of the biofilm, leaving all other material considerations aside [40]. Hydrophobic coatings will nonetheless show a more significant reduction for biofilm attachment, especially at a high shear flow force, than hydrophilic ones within the piping system, even if the critical effect would be carried by the surface materials themselves. Additionally, the biofilm attachment preference is non-selective.

#### 3.1.3. Physical Constraints for the Attached Cell

Physical restrictions of the biofilm cells are thought to function as another crucial component for the adhesion on the surface materials, in addition to the thermodynamic and surface energy parameters, particularly for the close proximity to surfaces and adjacent cells. Holonomic constraints and non-holonomic constraints are the two categories into which physical limitations fall. Independent of the dynamical states of the system, the space-time geometry of the field to which the system belongs imposes holonomic limitations. In contrast to activities occurring within a cell, which are significantly influenced by gravitational/magnetic force, studies carried out in the absence of gravity [40] show that gravitational or magnetic fields are great instances of this class of restrictions. Contrarily, non-holonomic restrictions must be continuously restored because they alter during biological activities. Self-organizing systems are those with such restrictions, in which dynamics act on the constraints to reproduce them [41]. These two constraints can be spatially limited and contingent, meaning that their existence is constrained to a specific period, as is the case during some developmental phases, or they can have a hierarchical distribution, which means that they can span across various levels and result in morphological changes, as previously described by Pattee [42]. According to the concept of a hierarchy of constraints, the dynamics of the lower level cannot be simply averaged out since the upper-level constraints place a special limitation on it [43]. 

### 3.2. Bacterial Surface Sensing and Adhesion

#### 3.2.1. EPS Production and Formation of Conditioning Layer

**Figure 3:**
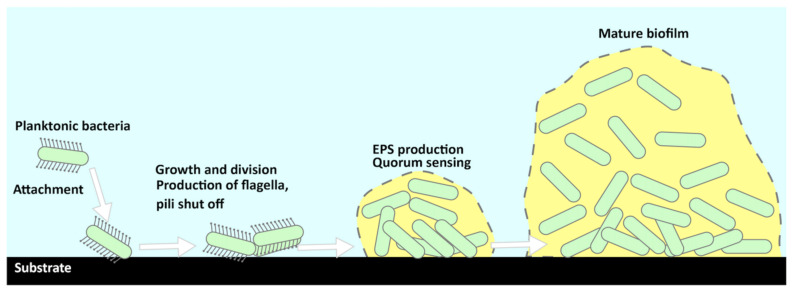
Bacteria attachment on a surface, EPS production, and mature biofilm structure [45].

The bacterial community within the EPS is shielded by biofilms from external stressors such as the nearby environment, predatory microorganisms, and antibiotics. The resultant increase in resistance to antibiotics in biofilms can be attributed to (i) genetic exchanges via horizontal gene transfer resulting in upregulation of biofilm-specific ABR genes, (ii) the diffusion barrier for antimicrobial molecules, and (iii) dormant persister cells. The layer of protein deposited by a protein found in the environment or bacteria itself may form during the growth of the biofilm. Secondary signaling molecules (such as Cyclic di-GMP (c-di-GMP)) are catalyzed by coordinated bacterial gene transcriptional changes via the QS system, which downregulate motile appendages such as flagella and promote EPS production [44]. At this time, bacteria secrete bound EPS with tightly bound EPS (TB-EPS) sustaining groups together, and loosely bound EPS (LB-EPS) joining various bacteria clusters to build sturdy micro-colonies. The biofilm’s structural stability is provided by the bacterium community’s ongoing production of EPS, which is made up of protein, polysaccharides, eDNA, bacterial lytic products, and host chemicals [45]. The influencing layer, which consists of inorganic compounds, organic macromolecules, and cellular elements, has the ability to mask surface coatings such as ammonium salts [22,29,46]. This is problematic because the deposited layer would bypass the mechanisms used to hinder the formation of biofilm on the faces of industrial piping systems (Figure 3).

#### 3.2.2. DLVO Theory

The DLVO theory is a colloidal dispersion stability theory that explains the development of a repulsive force as two particles approach one another by using the zeta potential. Due to their close contact with a surface, bacteria may sense changes in pH via the protein outer membrane protein A (OmpA), osmolality, and flagella rotation, which alters the transcription of other genes and starts the attachment process. The DLVO hypothesis combines the Van Der Waals and Coulomb interactions [7,46], which includes the cell’s interaction with its surroundings (attractive forces of Van Der Waals and repulsive electrostatic Coulombs interactions). The forces of Van der Waals are weak, attractive non-covalent forces, while the interaction of Coulombs refers to the creation of an electrical double layer in an aqueous solution (often negatively charged). One strategy used by bacteria to overcome repellent forces is the application of a long-stranded eDNA to break through the negative electrical barrier. Surfaces that release chemicals may also encourage bacterial adhesion. N-Acetylglucosamine (GlcNAc) monomers and oligomers, which are a chemoattractant of V. cholerae, start migrating towards the source and attaching to the chitin surface, and are produced, for instance, during the degradation of chitin surfaces [47,48].

### 3.3. Phenotypes Change and Bio-Communication

#### 3.3.1. Bacteria Motility

Bacteria motility before attachment mostly relies on the appendages which end up with the phenotypic change of the biofilm. Either motile appendages (e.g., *pili* and *flagella*) or immotile appendages succeeded by Brownian motion (continuous random movement of particles in a fluid) may become the substratum materials for the attraction of the planktonic bacteria [49]. However, the bacteria with motile appendages are able to abide by a substratum with regard to flow velocity, while the bacteria with no motile appendages are only able to adhere to a substratum at a medium and low velocity.

#### 3.3.2. Quorum Sensing (e.g., *E. coli*)

Quorum sensing (QS) is a communication system between cells that controls the expression of genes based on the density of a cell [50]. QS controls many morphological expressions, including biofilm formation, luminescence, motility, and virulence [24]. The activation of genetic mechanisms to introduce changes in the transcription of a bacterial gene at a given concentration of a bacteria may be influenced by the quorum sensing mechanisms [51]. Bacterial ability and detection of environmental changes are mediated by QS signaling molecules. Microbial cells produce two distinct types of quorum sensing (QS) chemical signaling molecules. Gram-negative bacteria produce N-acyl-L-homoserine lactone (AHL), while gram-positive bacteria produce Autoinducer Peptides (AIP) [52,53] acting as the signaling molecule used on inter- and intra-levels of species, allowing communication between gram negative and positive bacteria. Even the bacteria that do not produce AHLs (e.g., *E. coli* and *Salmonella*) still have AHL response regulator protein to receive the signal molecules.

#### 3.3.3. Catalysing c-di-GMP

Cyclic di-GMP (c-di-GMP) catalyzation is a physiological and biochemical mechanism. The life cycle of biofilms depends on the dynamic intracellular signaling molecule bis-(3′-5′)-cyclic dimeric guanosine monophosphate (c-di-GMP). Given that c-di-GMP-modifying enzymes respond to changes in oxygen, nutrients, and other environmental signals, and that biofilms have physiological heterogeneity, the c-di-GMP concentration is likely to vary in different areas of the biofilms where the bacteria are exposed to various signals or nutrient concentrations [54]. It has not yet been proven that the distribution of c-di-GMP is heterogeneous. This is partly because biofilm extraction and homogenization are required by this present approach for detecting c-di-GMP, which is based on liquid chromatography-mass spectrometry (LC-MS) [55]. Since metabolic gradients cause spatial variation in the biofilm, concentrations are an average of the whole biofilm and do not take this into account [51,52]. Applying the Chemotaxis system, which uses membrane-embedded sensors to measure the concentration of extracellular small molecules and ions, is an advanced technology [53,55]. For the comparison of the signals’ inputs across time, and in order to address the c-di-GMP concentration in various areas of the biofilm, a signal report that can be calibrated to the c-di-GMP concentration could be used [54].

#### 3.3.4. Changes in Swarming and Biofilm Community Structure

Flagellar production and motility are both reduced in biofilm cells, as previously stated. c-di-GMP, which similarly upregulates the creation of adhesion elements, can have a negative effect on flagellar motility, both directly and through cellulose production [56,57,58]. Moreover, the upregulating infection-related genes may also attract host invasion in terms of swarming and biofilm community structure, e.g., certain bacteria use CRISPR-associated (Cas) proteins from (CRISPR)-CRISPR-associated (CRISPR-Cas) systems to concentrate on their own genes, thus altering virulence during the mammalian invasion. However, it remains unknown whether CRISPR-Cas systems in *Salmonella* perform likely operations during the invasion of host cells by bacteria. The virulence changes in *Salmonella enterica serovar Enteritidis* were proven to be caused by Cas3 deletion, after systematically examining the genes controlled by Cas3 in a type I-E CRISPR-Cas system; whereas, Cas3 is an ATP-dependent single-strand DNA (ssDNA) translocase/helicase enzyme that degrades DNA as part of CRISPR based immunity. In comparison to the Cas3 WT Salmonella strain, Cas3 deletion increased the lsrFGBE genes in the lsr (luxS regulated) operon related to quorum sensing (QS) and decreased biofilm-production-related genes and *Salmonella* pathogenicity island 1 (SPI-1) genes related to the Type 3 Secretion System (T3SS) [59]. To conclude, the changes in swarming and biofilm community structure may also influence the bio-communication via the physical, as well as a bio-chemical effect [60].

## 4. Practice for the Biofilm Removal of Industrial Piping Systems

### 4.1. Strategic Mechanism of Biofilm Removal 

**Figure 4:**
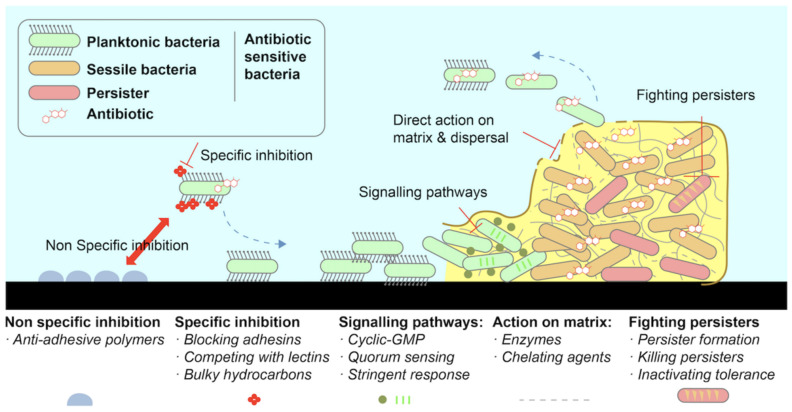
Different biological mechanisms to inhibit biofilm growth [9].

There are numerous biological mechanisms that can be used to prevent or remove the formation of biofilm on a surface. These include specific and non-specific inhibitors such as anti-adhesive polymers, enzymatic action on the extracellular polymeric substance (EPS) matrix, interfering signaling pathways, and persister destruction [9] (Figure 4). Photocatalytic and/or EPS degradation, quorum sensing, and super hydrophilic/hydrophobic surface structure are some of the possible biological mechanisms and surface-morphology mechanisms that could be used [9,24]. The use of enzymes to break down the EPS matrix secreted by the bacterial community, leaving the biofilm structure unstable and susceptible to external environmental forces and antibiotics, is known as EPS degradation [8]. Quorum sensing/quenching works by interfering with signaling pathways during signal molecule biosynthesis, degradation, and reception [9,55].

### 4.2. Oxidizing Biocides

Regarding industrial piping, biocides are commonly used in the industry to control biofilm [22]. This is comparable to the use of antibiotics in microbiology. Biocides are chemicals that are used to kill all living organisms in a water system [12]. In large-scale water disinfection applications, traditional oxidizing biocides such as hydrogen peroxide and sodium hypochlorite (bleach) are commonly used [19,20]. These chemicals, however, can present significant operational challenges. The bacteria in hydrogen peroxide release two enzymes, namely KatA and KatB. Their purpose in this study was to neutralize the biocide. Biofilms are nonetheless comparatively robust to hydrogen peroxide even in the absence of catalase function [61].

Another corrosive and highly oxidizing biocide for biofilm removal is bleach, but it can cause accelerated corrosion of metal components within the industrial plant system, such as heat exchangers and pipes. Improper bleach use can result in significant costs, such as a reduced expected life of equipment and increased maintenance. Furthermore, sodium hypochlorite has the ability to oxidize metals. The oxidation of the metal can be improved by removing oxidized metal through clarification or filtration. However, if the oxidized metal is not removed, it can accumulate on the metal surface, resulting in the formation of differential inflation cells, which promote corrosion. Furthermore, as microorganism nutrients, oxidized metals can exacerbate microbial corrosion. The main metals involved in these corrosion mechanisms are iron and manganese. The effect of bleaching agents does not last long enough in large circulating systems [62]. To allow sufficient residual bleach in the rest of the system, an excessive amount of bleach may be required in the rest of the system. Both biocides, as strong oxidizing agents, react quickly with organic matter and metals in the system, reducing their germicidal ability. This can result in varying amounts of bleach residue throughout the plant [54,55].

### 4.3. Detergent and Enzymes Cleaner

Since mechanical devices are difficult to reach, the use of high temperatures or harsh chemicals and long, narrow endoscope channels could damage the sensitive components built into endoscopes. Therefore, mild cleaning elements are required to counter biofilms in order to reprocess endoscopes. Therefore, detergent is used to weaken the EPS of the biofilm, which contain extracellular DNA, lipids, polysaccharides, and other substances. Enzymes such as cellulase, protease, D-Nase I, and amylase have been observed to aid in the removal of this biofilm. In conclusion, the efficiency of biofilm detachment can be improved by including enzymes in cleaning agents [22,25]. 

Furthermore, the use of the enzyme benzonase could potentially have outstanding results based on its broad range of particularity—reducing all forms of RNA and DNA—as well as its ability to operate in conditions with a wide temperature and pH range [39]. Since enzymes easily become inactive, immobilization may be beneficial as it allows more enzymes to become stable, efficient, and more active. Kim et al. demonstrated that by inactivating acylase on a surface, there was a possibility of preventing the formation of biofilm and maintaining a higher percentage of enzymatic activity in nanofiltration over a duration of 20 washing and reaction cycles [34].

### 4.4. Anti-Adhesion Coating

Since the microorganisms in the biofilm are more tolerant to the disinfectant, it is, therefore, desirable to develop coating compositions that can be applied to many surfaces and will continue to control microbial contamination for a longer period of time. It is also desirable to have a removable coating composition that will allow for easy removal of the coating. Anti-adhesion coatings prevent the early-stage formation of biofilm, which should be preferred by several organizations within the industry [19,22]. The four element properties of the anti-adhesion covering surface include (i) reactivity and chemical composition, (ii) hydrophobicity and hydrophilicity, and (iii) surface charge and (iv) texture or surface roughness. Below is an introduction of promising use with the change of each of the four properties [63]:

- Trimethylsilane plasma nanocoatings can be used with low-temperature plasma covering technology to coat faces of titanium and stainless steel to reduce the formation of adherence of S. epidermidis biofilm. The hindrance of such biofilm could be due to the covering coating’s smoothness, low surface energy, chemical inertness, and surface-bound methyl groups. The changed face characteristics could cause less protein adsorption to the covered surfaces.
- A mixture of both low surface energy fluorinated silane xerogel and nanostructured silica colloids were used to form a superhydrophobic covering on glass. According to the findings, the rate of adsorption of fibrinogen was low on the superhydrophobic surface, thus resulting in weak attachment of S. aureus. However, to achieve reduced bacterial attachment and protein adsorption levels, a low nano-textured shape and facet energy chemistry of the superhydrophobic coating can be used.
- The surface roughness compared to smooth surfaces featured significantly increased development and adherence of biomaterials. On the contrary, S. aureus cells were strongly attached to mechanical titanium that had been chemically polished as compared to the titanium that had just been received despite the polished surfaces being smoother [16].

## 5. Conclusions and Positions

Bacterial adhesion study necessitates a multidisciplinary approach that includes recognizing the characteristics of surfaces identified by bacteria, conducting a systematic study of the molecular mechanisms and biochemical responses bacteria use when sensing surfaces, and comprehending surface manipulation to achieve the desired cellular response. Furthermore, surface mRNA analysis using fluorescent technology to demonstrate gene expression as well as assessing the chemical composition of individual cells indicates their importance in the biofilm attachment and growth process.

In terms of biofilm inhibition, conditioning layer prevention is still one of the first methods to be considered. Chemical control methods such as the structural properties of surfaces inspired by slippery, liquid-infused porous facets, low effective stiffness nanostructured surfaces, and natural materials such as lotus leaves and shark skin, remain the most viable methods for industrial piping systems. In addition, these preventive methodologies and approaches can be used in agriculture, biomedicine, food safety, and dentistry.

## Figures and Tables

**Table 1:** Summary for the cell attachment mechanism and theory.

Attachment Process	Findings and Positions
Regions in the fluid that motile bacteria occupy	1. Bulk liquid—no effect on cells; 2. Near surface bulk liquids—cells experience hydrodynamic (shear) effects; 3. Near surface constrained—cells experience hydrodynamic (shear) and physicochemical effects (e.g., Van der Waals & electrostatic forces).
Attachment to surfaces	1. Non-motile bacteria—able to adhere at low and moderate fluid velocities but not high velocities; 2. Motile bacteria—able to adhere to surfaces regardless of fluid velocities.
Bacterial density buoyance	Sedimentation of bacteria that increases in stationary phase (exception of *Vibrio parahaemolyticus*).

## Data Availability

Not available.
